# Acute and Subacute Toxicity of Safranal, a Constituent of Saffron, in Mice and Rats

**Published:** 2013

**Authors:** Hossein Hosseinzadeh, Saied Sadeghi Shakib, Abbas Khadem Sameni, Elahe Taghiabadi

**Affiliations:** a *Pharmaceutical Sciences Research Center, Department of Pharmacodynamy and Toxicology, School of Pharmacy, Mashhad University of Medical sciences, Mashhad, IR. Iran.*; b *Department of Pharmacodynamy and Toxicology, School of Pharmacy, Mashhad University of Medical sciences, Mashhad, IR. Iran. *; c *Javadolaemeh Cardiac Hospital, Mashhad, I. R. Iran. *

**Keywords:** Safranal, *Crocus sativus*, Saffron, Acute toxicity, Subacute toxicity.

## Abstract

The acute and sub-acute toxicity of safranal were studied in rat and mice within 2 and 21 days after exposure, respectively. For subacute toxicity, changes in weight as well as biochemical, hematological and pathological parameters were studied. The intraperitoneal LD_50_ values of safranal were 1.48 mL/kg in male mice, 1.88 mL/kg in female mice and 1.50 mL/kg in male rats. Oral LD_50_ values were 21.42 mL/kg in male mice, 11.42 mL/kg in female mice and 5.53 mL/kg in male rats. For subacute toxicity, safranal was administered orally to male rats once daily for 21 days. In hematological tests, a significant decrease in RBC counts, hematocrit, hemoglobin and platelets were observed. Safranal decreased cholesterol, triglyceride and alkalin phosphatase. Lactate dehydrogenase and serum urea nitrogen were increased by safranal. Histological studies indicated that safranal did not have any toxic effect on the heart, liver and spleen. However, pathological changes were seen in the kidney and lung. According to LD_50_ values, safranal was low-toxic in acute intraperitoneal route and practically non-toxic in acute oral administration in both mice and rats. In subacute toxicity, safranal changed some hematological and biochemical parameters.

## Introduction

*Crocus sativus *L. (saffron), a culinary spice, is utilized for its flavouring, colouring, aromatic and medicinal effects (1-3). Saffron quality depends on the presence of its three major constituent in its stigmas. Picrocrocin (4-(*β*-D-glucopyranosyloxy)-2, 6, 6-trimethyl- 1-cyclohexene-1-carboxaldehyde, C16H26O7), a monoterpene glycoside, is the main bitter principle of saffron. Safranal (2, 6, 6-trimethyl-1, 3-cyclohexadiene-1-carboxaldehyde), a monoterpene aldehyde, is a main constituent of the essential volatile oil responsible for saffron odor and aroma. Picrocrocin releases the aglycone, by *β*-Glucosidase action which is changed to safranal through the drying procedure and also is formed during the storage of saffron (2, 4-6). Crocin (glucosyl ester of crocetin) is responsible for colouring properties of saffron (2, 7-9). 

Safranal has shown different pharmacological activities such as bronchodilation (10, 11), reduction of ischemia-reperfusion injury in kidney (12), brain (13, 14 ) or skeletal muscle (15), prevention of gentamicin- or hexachlorobutadiene-induced nephrotoxicity (16, 17)**, **reduction of subacute toxicity of diazinon (18), hypnotic and antianxiety (19), hypotensive (20), anticonvulsant (21-23), antidepressant (24), genoprotective (25), antioxidant (26, 27), antinociceptive (28), antitussive (29) and anticancer activities (30). 

The LD_50_ values of saffron stigma and petal extracts by intraperitoneal administration in mice were 1.6 and 6 g/kg, respectively. In a subacute toxicity study, both extracts decreased the value of hematocrit, hemoglobin and erythrocytes, however, the stigma extract did not cause any significant pathological effects in different organs (30). In humans, saffron with doses between 1.2 and 2 g induced nausea, vomiting, diarrhea and bleeding (32). High oral and intraperitoneal doses of crocin (3 g/kg) did not cause death within 2 days of study in mice (33). At high doses (200 and 400 mg/day), saffron tablets changed some hematological and biochemical parameters in healthy adult volunteers. However, these changes were in normal ranges and they were not clinically important (34). Despite the wide use of saffron in many countries as an herbal remedy and the useful pharmacological effects of safranal, few studies have been published in the literatures about their toxicological profiles. Therefore, the aims of the present study were to assess the acute and subacute oral and intraperitoneal toxicity of safranal in rodents. 

## Experimental

*Chemicals*

Safranal was purchased from Fluka Chemie AG (Buchs, Switzerland). Enzymatic reagent kits for determination of alanine aminotransferase (ALT), aspartate aminotransferase (AST), lactic acid dehydrogenase (LDH) and creatine phosphokinase (CPK) were purchased from Greiner Bio-One and alkaline phosphatase, bilirubin, blood urea nitrogen (BUN), albumin, cholesterol and triglyceride were purchased from Pars Azmoon Co. All other chemicals and solvents used throughout this study were of analytical grade.

*Animals*

Male Wistar rats (weighing approximately 200–250 g) and BALB/c mice of both sexes (weighing approximately 25–30 g) were obtained from the animal house of the Pharmaceutical Sciences Research Center of Mashhad University of Medical Sciences, Mashhad, Iran. Animals were housed in a colony room under a 12/12 h light/dark cycle at 21 ± 2°C and had free access to water and food. All animal experiments were approved by the Animal Care Committee of Mashhad University of Medical Sciences. 

*Acute toxicity of safranal in mice and rats*

Animals randomly were divided into several groups (n = 6/sex). The first group (control group) received saline and other groups were treated with different doses of safranal by oral or intraperitoneal route. Following administration, animals were observed for signs of toxicity and mortality for a period of 48 h after treatment. The lethal dose (LD_50_) was estimated according to the method described by Litchfield and Wilcoxon method (PHARM/PCS software version 4).

*Subacute toxicity*

Twenty four male rats were randomly divided into four groups (n = 6). Animals were given saline as a control. According to LD_50_ value, animals received safranal at the doses of (0.1, 0.25 or 0.5 mL/kg/day, orally) for 21 days. The weight of the body was determined weekly. Animals were observed for general behavioral and signs of abnormalities during the experiment duration. 

*Blood sampling*

After 21 days, animals were anaesthetized by chloroform. Blood samples were collected by cardiac puncture into sterile tubes with anticoagulant (EDTA) for hematological tests and without anticoagulant tubes for biochemical tests. Blood samples (without anticoagulant) tubes were centrifuged at 5000 rpm for 15 min and serum was separated. 

*Hematological and biochemical analyses*

For hematological analysis, white blood cell (WBC), red blood cell (RBC), hemoglobin concentration, hematocrit, mean corpuscular volume (MCV), mean corpuscular hemoglobin (MCH), mean corpuscular hemoglobin concentration (MCHC) and platelets were measured using an automatic hematocyte analyzer. 

The serum was analyzed for alkaline phosphatase (ALP), aspartate aminotransferase (AST), alanine aminotransferase (ALT), lactic acid dehydrogenase (LDH), creatine phosphokinase (CPK), total bilirubin, serum glucose, total cholesterol, triglyceride, albumin, serum urea nitrogen (BUN), creatinine using commercial colorimetric kits.

*Histopathological analysis*

After blood collection for hematological and biochemical tests, organ tissues like heart, liver, kidneys, spleen and lungs were carefully removed for histological tests. After macroscopic study, these organs were fixed in a 10% buffered formalin solution. The prepared sections were subjected to hematoxylin–eosin for histopathological observations under an optical microscope. 

*Statistical analysis*

Data were determined as mean ± SEM. All data were analyzed using analysis of variance (ANOVA) followed by Tukey- kramer. Statisticsl significance was defined as the p-values less than 0.05 (p < 0.05). 

## Results

*Acute toxicity studies*

The intraperitoneal LD_50_ values of safranal in male and female mice were 1.48 mL/kg (0.96- 2.29) and 1.88 mL/kg (1.03- 3.44), respectively and the maximum non-fatal dose for both sexes was 0.75 mL/kg. The oral LD_50_ values of safranal in male and female mice were 21.42 mL/kg (7.4- 64.29) and 11.42 mL/kg (7.44- 17.53), respectively and the maximum non-fatal dose for female mice was 5 mL/kg. The intraperitoneal and orally LD_50_ values of safranal in male Wistar rats were 1.5 mL/kg (1.06- 2.13) and 5.53 mL/kg (3.68- 8.3), and the maximum non-fatal doses were 0.75 mL/kg and 3.5 mL/kg, respectively. 

The adverse effects and lethality increased progressively with increasing doses by both routes. All of the mice and rats died after receiving doses of 5 and 7 mL/kg intraperitoneally and deaths occurred within 2 days. In mice and rats, safranal interaperitoneally did not cause any lethality at doses of 0.5 and 0.75 mL/kg.

*Oral subacute toxicity*

**Figure 1:**
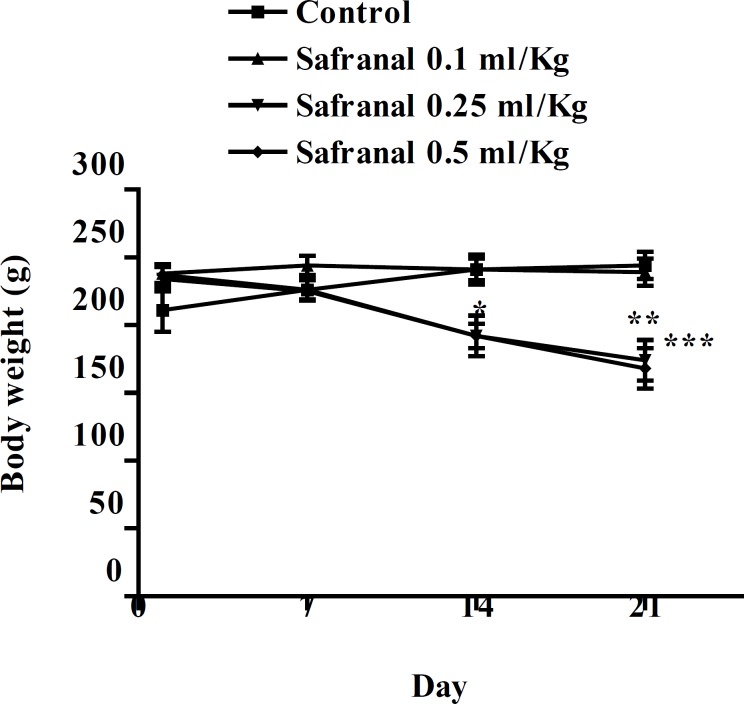
Body weight gain curves of male Wistar rats treated orally with safranal (0.1, 0.25 and 0. 5 mL/kg) via oral route for 21 consecutive days. The values are expressed as mean ± SEM. (n= 6). ***p < 0.001, **p < 0.01, compared with control. Tukey–Kramer

After approximately 5-7 min of treatment, animals became excited and exhibited hyperactivity; this was followed by sedative effects, relaxation and decrease in locomotor activity. Also asthenia, anorexia, decrease in food and water consumption and weight loss were observed after the oral treatment, and were more significant at higher doses (figure 1). At the end of the experiment, the body weights of animals were significantly decreased.

As shown in Table 1, RBC, hemoglobin, hematocrit, and platelets were significantly decreased in all doses of treatment groups compared to the control group, however the hematological analysis showed no significant difference of MCV, MCH, MCHC and WBC in treated groups in relation to the control. 

**Table 1:** Effect of orally administration of safranal at doses 0.1, 0.25 and 0.5 mL/kg on hematological parameters in male Wistar rats treated for 21 consecutive days.

**Parameter**	**Control**	**Safranal ** **0.1 mL/Kg**	**Safranal ** **0.25 mL/Kg**	**Safranal ** **0.5 mL/Kg**
WBC (×10^3^μL)	6.13 ± 0.82	6.1 ± 0.37	7.6 ± 0.57	5.75 ± 0.94
RBC (×10^6^/μL)	8.23 ± 0.17	7.5 ± 0.21*	4.94 ± 0.19 ***	3.7 ± 0.2 ***
Hemoglobin (g /dL)	15.28 ± 0.23	13.18 ± 0.33	8.56 ± 0.33	6.05 ± 0.46 ***
Hematocrit (%)	44.7 ± 1.02	37.86 ± 0.94 ***	26.58 ± 0.72 ***	22.66 ± 0.71 ***
MCV (fL)	54.03 ± 0.53	50.5 ± 0.3	51.25 ± 1.51	51.85 ± 1.11
MCH (pg)	18.63 ± 0.22	17.61 ± 0.13	17.91 ± 0.37	18.06 ± 0.21
MCHC (g/dL)	34.53 ± 0.26	34.85 ± 0.11	34.06 ± 0.46	35.26 ± 0.3
Platelets (×10^3 ^/μL)	910 ± 41.36	689.33 ± 76.3 *	269.16 ±38.78***	84.66 ± 11.56***

Data showed as mean ± SEM. (n = 6). ***p < 0.001, *p < 0.05, compared with control. Tukey–Kramer. RBC: red blood cell, MCV: mean corpuscular volume, MCH: mean corpuscular hemoglobin, MCHC: mean corpuscular hemoglobin concentration and WBC: white blood cell.

The subacute oral administration of safranal (daily for 21 days) did not show any significant difference in some biochemical profiles such as serum glucose, total bilirubin, serum creatinine, albumin, aspartate aminotransferase (AST), alanine aminotransferase (ALT), creatine phosphokinase (CPK) and total bilirubin (Tables 2). However, the levels of total cholesterol, triglyceride showed a significant reduction in all doses and alkalin phosphatase (ALP) was decreased in animal given 0.5 and 0.25 mL/kg of safranal. Safranal induced an increase in the level of lactic acid dehydrogenase (LDH) and serum urea nitrogen (BUN) in the treated group at high dose (0.5 mL/Kg) (Tables 2).

**Table 2:** Effect of orally administration of safranal at doses 0.1, 0.25 and 0.5 mL/kg on serum biochemical parameters in male Wistar rats treated for 21 consecutive days.

**Parameter**	**Control**	**Safranal ** **0.1 mL/Kg**	**Safranal ** **0.25 mL/Kg**	**Safranal ** **0.5 mL/Kg**
Glucose (mg/dL)	145.5 ± 10.93	127.83 ± 37.41	152 ± 10.35	154.16 ± 20.71
BUN (mg/dL)	35.66 ± 1.3	36.66 ± 0.66	37.5 ± 2.89	73.33 ± 14.22 **
Creatinine (mg/dL)	0.43 ± 0.02	0.45 ± 0.02	0.38 ± 0.03	0.36 ± 0.2
Cholesterol (mg/dL)	59.33 ± 2.48	62 ± 2.43	46.33 ± 2.1 **	37.5 ± 1.45 ***
Triglycerides (mg/dL)	74.33 ± 8.89	45 ± 4.34 **	43.66 ± 4.23 **	40.33 ± 4.16 **
LDH (IU/L)	248.16 ± 59.05	210.83 ± 27.4	211.5 ± 28.78	434.66 ± 50.77 *
CPK (IU/L)	371 ± 84.8	290.5 ± 26.55	533.2 ± 55.13	554.6 ± 114.59
ALT (IU/L)	69.16 ± 6.41	70 ± 6.41	58.16 ± 3.04	169.33 ± 134.16
AST (IU/L)	87.66 ± 9	99.33 ± 7.94	117.5 ± 14.24	123.4 ± 40.15
ALP (IU/L)	65.75 ± 9.28	63.66 ± 3.52	35.2 ± 2.69 **	21 ± 3.96 ***
Albumin (g/dL)	3.55±0.07	3.5±0.05	3.31±0.09	3.51 ± 0.14
Total bilirubin (mg/dL)	0.45 ± 0.02	0.38 ± 0.01	0.38 ± 0.03	0.36 ± 0.02

Data showed as mean±SEM. (n =6). ***p < 0.001, **p < 0.01, *p < 0.05, compared with control. Tukey–Kramer,. BUN: blood urea nitrogen, AST: aspartate aminotransferase, ALT: alanine aminotransferase, LDH: lactic acid dehydrogenase, CPK: creatine phosphokinase and ALP: alkaline phosphatase

Pathological examinations of the tissues indicated that there were no detectable abnormalities and alterations in the microscopic examination of the organs such as heart, liver and spleen in comparison to the control. However, histological examinations studies indicated abnormalities and toxic effects in kidneys and lung, especially at a higher dose (0.5 mL/Kg). Edema and cytolysis were noticed in the kidney and progressed emphysema and lymphocyte infiltration were observed in lungs.

## Discussion

Saffron and its constituents are widely used as spice and medicinal plant in folk medicine. Medicinal herbs usually have different constituents that possess the potential to cause useful and/or harmful effects. To evaluate the appropriate effect of safranal and for its further development as a therapeutic medicine, we needed to assess both acute and subacute toxicity for this compound. The results of the acute toxicity study indicated that LD_50_ values of safranal varied according to species and route of administration. According to the toxicity classification, substances with a LD_50_ value within the range of 1–5 g/kg are considered a practically low-toxic and substances that show LD_50_ higher than 5.0 g/kg may be considered practically non-toxic (35, 36). The calculated safranal LD_50_ values of intraperitoneal route in male and female mice and male Wistare rats were in the range of 1–5 g/kg. Thus, safranal should be considered as practically low-toxic in acute intraperitoneal route. In regard to LD_50_ values in mice and rats by the oral route (> 5.0 g/kg), the present investigation shows that safranal is practically non-toxic in acute oral administration. There were no sex-related differences in the toxicity of safranal (p > 0.05). Due to variations in systems of detoxification and differences in absorption, distribution, excretion mechanisms in different animal species, there are species differences in response to toxic substances (37). Safranal was more toxic when administrated intraperitoneal as compared to the oral route. The reason for the high toxicity of safranal by the intraperitoneal route compared to the oral administration, might be due to more first pass effect and lower rate of absorption with the oral treatment.

In the subacute toxicity study, safranal at doses of 0.1, 0.25 and 0.5 mL/kg by oral rout for 21 days did not cause any death in rats. However, treated animals showed some evidence of toxicity. Behavioral changes such as hyperactivity and excitation after 5-7 min of treatment might be related to the irritant nature of most essential oils.

Safranal showed sedative effects and reduction of locomotor activity. Safranal has an interaction with GABA _A_ receptors (22) and it was demonstrated that safranal has hypnotic and anti anxiety effects (19). 

The subacute tests indicated a significant decrease in body weight. It may be due to safranal effect on reduction of appetite which leads to a reduced food consumption. The daily supplementation over 8 weeks with a food supplement of a patented extract of saffron stigma significantly reduced the frequency of snacking events and a slight body weight loss in healthy women was observed (38).

In this study, the results of the hematological parameters showed a significant decrease of RBC, hemoglobin, hematocrit, and platelets in the treated groups as compared with the control group. Our results are quite similar to results of other investigating groups that indicated the reduction of hemoglobin and hematocrit levels and total RBC count by saffron (31, 39).

In addition, the treatment with safranal did not alter the biochemical parameters, except for an increase in lactic acid dehydrogenase (LDH) and serum urea nitrogen (BUN) levels in treated groups and decrease in total cholesterol, triglyceride and alkaline phosphatase (ALP). Pathological evaluations of the tissues did not indicate changes and abnormalities in organs except in kidney and lungs.

In this study, there were no changes on the common markers of liver toxicity such as levels of the liver enzymes ALT and AST and bilirubin. Thus, it may be presumed that safranal did not cause significant damage to this organ. The reduction of alkaline phosphatase level might be related to malnutrition and decrease in food and water consumptions and might not be attributed to hepatotoxicity. 

Safranal treated rats showed an increase on blood urea nitrogen (BUN). The levels of BUN and creatinine are good indicators of renal function. The increased level of BUN in the treated groups can be attributed to renal damage and this is further confirmed by the pathologic findings of this organ. However, in this study no difference in levels of serum creatinine was observed. 

The levels of blood cholesterol and triglyceride significantly reduced in the tested groups, as compared to the control group, indicating that safranal has hypolipidemic effects. In addition, the reduction of blood cholesterol and triglyceride might be related to decrease in body weight, diminished food and malnutrition.

The data showed no change in the level of creatine phosphokinase (CPK) in control and all administrated groups. The creatine phosphokinase (CPK) is one of the indicators available for the diagnosis of cellular damage to the heart (40, 41). No significant changes in creatine phosphokinase (CPK) values and no histopathologic damage in the heart indicates that the sub-acute treatment of safranal at the above mentioned doses did not induce any damage to the heart. The level of lactic acid dehydrogenase (LDH) was increased in the treated group at the high dose (0.5 mL/Kg). Since many tissues contain LDH, elevated total LDH is considered a nonspecific indication of cellular damage.

The results of the previous study on acute and subacute toxicity of crocin indicated that crocin at pharmacological doses did not demonstrate any mortality and damages to all the major organs of the body (33) but in this study safranal showed mortality in mice and rats as well as abnormalities in kidney and lungs and also safranal induced decrease in hematological paremeters such as RBC and hemoglobin. With regards to these data, safranal showed more toxicity than crocin.

In conclusion, this study presented the results on the acute and subacute toxicity of safranal, a constituent of *Crocus sativus *that can be very useful for any future *in-vivo *and clinical studies. According to the LD_50_ data, safranal was low-toxic in acute intraperitoneal route and practically non-toxic in acute oral administration in both mice and rats. The data suggest that the oral administration of safranal, in subacute toxicity test did not induce any toxic effects in many organs except for kidneys and lungs. However, further long-term and also other routs of administration studies are necessary to re-evaluate these results. 

## References

[1] Carmona M, Sanchez A, Ferreres F, Zalacain A, Tomas-Barberan F, Alonso GL (2007). Identification of the flavonoid fraction in saffron spice by LC/DAD/MS/MS: Comparative study of samples from different geographical origins. Food Chem.

[2] Lage M, Cantrell CL (2009). Quantification of saffron (Crocus sativus L) metabolites crocins, picrocrocin and safranal for quality determination of the spice grown under different environmental Moroccan conditions. Sci. Hortic.

[3] Melnyk JP, Wang S, Marcone MF (2010). Chemical and biological properties of the world›s most expensive spice: Saffron. Food Res. Int.

[4] Zougagh M, Rios A, Valcarcel M (2006). Determination of total safranal by in situ acid hydrolysis in supercritical fluid media: Application to the quality control of commercial saffron. Anal. Chim. Acta.

[5] Maggi L, Carmona M, Zalacain A, Kanakis CD, Anastasaki E, Tarantilis PA, Polissiou MG, Alonso GL (2010). Changes in saffron volatile profile according to its storage time. Food Res. Int.

[6] Maggi L, Sanchez AM, Carmona M, Kanakis CD, Anastasaki E, Tarantilis PA, Polissiou MG, Alonso GL (2011). Rapid determination of safranal in the quality control of saffron spice (Crocus sativus L). Food Chem.

[7] Pintado C, Miguel A, Acevedo O, Nozal L, Novella JL, Rotger R (2011). Bactericidal effect of saffron (Crocus sativus L) on Salmonella enterica during storage. Food Contr.

[8] Papandreou MA, Tsachaki M, Efthimiopoulos S, Cordopatis P, Lamari FN, Margarity M (2011). Memory enhancing effects of saffron in aged mice are correlated with antioxidant protection. Behav. Brain Res.

[9] Shatia AA, Elsaid FG, Hafeza EE (2011). Biochemical and molecular aspects of aluminium chloride-induced neurotoxicity in mice and the protective role of Crocus sativus L. extraction and honey syrup Cell. Mol. Neurosci.

[10] Boskabady MH, Aslani MR (2006). Relaxant effect of Crocus sativus (saffron) on guinea-pig tracheal chains and its possible mechanisms. J. Pharm. Pharmacol.

[11] Nemati H, Boskabady MH, Ahmadzadeh Vostakolaei H (2008). Stimulatory effect of Crocus sativus (saffron) on beta2-adrenoceptors of guinea pig tracheal chains. Phytomed.

[12] Hosseinzadeh H, Sadeghnia HR, Ziaee T, Danaee A (2005). Protective effect of aqueous saffron extract (Crocus sativus L) and crocin, its active constituent on renal ischemia-reperfusion-induced oxidative damage in rats. J. Pharm. Pharmacol. Sci.

[13] Hosseinzadeh H, Sadeghnia HR (2005). Safranal, a constituent of Crocus sativus (saffron), attenuated cerebral ischemia induced oxidative damage in rat hippocampus. J. Pharm. Pharm. Sci.

[14] Khalili M, Roghani M, Ekhlasi M (2009). The Effect of Aqueous Crocus sativus L. Extract on Intracerebroventricular Streptozotocin-induced Cognitive Deficits in Rat: a Behavioral Analysis. Iranian J. Pharm. Res.

[15] Hosseinzadeh H, Modaghegh MH, Saffari Z (2009). Crocus sativus L (saffron) extract and its active constituents (crocin and safranal) on ischemia-reperfusion in rat skeletal muscle. eCAM.

[16] Boroushaki MT, Mofidpour HB, Sadeghnia HR (2007). Protective effect of safranal against hexachlorobutadiene-induced nephrotoxicity in rat. Ir. J. Med. Sci.

[17] Boroushaki MT, Sadeghnia HR (2009). Protective effect of safranal against gentamicin-induced nephrotoxicity in rat. Ir. J. Med. Sci.

[18] Timcheh Hariri A, Moallem SA, Mahmoudi M, Memar B, Hosseinzadeh H (2010). Sub-acute effects of diazinon on biochemical indices and specific biomarkers in rats: Protective effects of crocin and safranal. Food Chem. Toxicol.

[19] Hosseinzadeh H, Noraei NB (2009). Anxiolytic and hypnotic effect of Crocus sativus aqueous extract and its constituents, crocin and safranal, in mice. Phytother. Res.

[20] Imenshahidi M, Hosseinzadeh H, Javadpour Y (2010). Hypotensive effect of aqueous saffron extract (Crocus sativus L) and its constituents, safranal and crocin, in normotensive and hypertensive rats. Phytother. Res.

[21] Hosseinzadeh H, Talebzadeh F (2005). Anticonvulsant evaluation of safranal and crocin from Crocus sativus in mice. Fitoterapia.

[22] Hosseinzadeh H, Sadeghnia HR (2007). Protective effect of safranal on pentylenetetrazol-induced seizures in the rat: Involvement of GABAergic and opioids systems. Phytomed.

[23] Sadeghnia HR, Cortez MA, Liu D, Hosseinzadeh H, Snead 3rd OC (2008). Antiabsence effects of safranal in acute experimental seizure models: EEG and autoradiography. J. Pharm. Pharm. Sci.

[24] Hosseinzadeh H, Karimi G, Niapoor M (2004). Antidepressant effects of Crocus sativus stigma extracts and its constituents, crocin and safranal, in mice. Acta Hortic.

[25] Hosseinzadeh H, Sadeghnia HR (2007). Effect of safranal, a constituent of Crocus sativus (saffron), on methyl methanesulfonate (MMS)-induced DNA damage in mouse organs: an alkaline single-cell gel electrophoresis (comet) assay. DNA Cell Biol.

[26] Hosseinzadeh H, Shamsaie F, Mehri S (2009). Antioxidant activity of aqueous and ethanolic extracts of Crocus sativus L stigma and its bioactive constituent, crocin and safranal. Pharmacog. Mag.

[27] Firouzi M, Moshayedi P, Sabouni F, Sabouni F, Keshavarz M (2004). The effect of crocin (a derivative of Crocus sativus L) on neural development and regeneration of rat: in-vivo and in-vitro study. Iranian J. Pharm. Res.

[28] Hosseinzadeh H, Shariaty VM (2007). Anti-nociceptive effect of safranal, a constituent of Crocus sativus (saffron), in mice. Pharmacologyonline.

[29] Hosseinzadeh H, Ghenaati J (2006). Evaluation of the antitussive effect of stigma and petals of saffron (Crocus sativus) and its components, safranal and crocin in guinea pigs. Fitoterapia.

[30] Escribano J, Alonso GL, Coca-Prados M, Fernández JA (1996). Crocin, safranal and picrocrocin from saffron (Crocus sativus L) inhibit the growth of human cancer cells in vitro. Cancer Lett.

[31] Karimi G, Tabibi N, Hosseinzadeh H, Shirzad F (2004). Sub-acute toxicity of saffron (Crocus sativus L) stigma and petal in rats. J. Med. Plants.

[32] Schmidt M, Betti G, Hensel A (2007). Saffron in phytotherapy: Pharmacology and clinical uses. Wien. Med. Wochenschr.

[33] Hosseinzadeh H, Shariaty MV, Khadem-Sameni A, Vahabzadeh M (2010). Acute and sub-acute toxicity of crocin, a constituent of Crocus sativus L (saffron), in mice and rats. Pharmacologyonline.

[34] Modaghegh MH, Shahabian M, Esmaeili H, Rajbai O, Hosseinzadeh H (2008). Safety evaluation of saffron (Crocus sativus) tablets in healthy volunteers. Phytomed.

[35] Loomis T (1968). Essential of Toxicology.

[36] Kennedy GL, Ferenz RL, Burgess BA (1986). Estimation of acute oral toxicity in rats by determination of the approximate lethal dose rather than the LD50. J. Appl. Toxicol.

[37] Piyachaturawat P, Tubtim C, Chuncharunee A, Komaratat P, Suksamrarn A (2002). Evaluation of the acute and subacute toxicity of a choleretic phloracetophenone in experimental animals. Toxicol. Let.

[38] Gout B, Bourges C, Paineau-Dubreuil S (2010). Satiereal, a Crocus sativus L extract, reduces snacking and increases satiety in a randomized placebo-controlled study of mildly overweight, healthy women. Nut. Res.

[39] Mohajeri D, Mousavi G, Mesgari M, Doustar Y, Khayat Nouri M (2007). Subacute toxicity of Crocus sativus L (Saffron) sigma ethanolic extract in rats. Am. J. Pharmacol. Toxicol.

[40] Engstrom R, Ramazzotto LJ, Hart R (1973). Cryoprotection in hearts as measured by CPK. Cryobiol.

[41] Seabra-Gomes R, Ganote CE, Nayler WG (1975). Species variation in anoxic-induced damage of heart muscle. J. Mol. Cell. Cardiol.

